# Post-operative optical coherence tomography angiography features of chorioretinal folds resulting from pleomorphic adenoma of the lacrimal gland (PALG) of orbit- a case report

**DOI:** 10.1186/s12886-020-01750-0

**Published:** 2020-12-10

**Authors:** Nan-Ni Chen, Chien-Hsiung Lai, Tsai Yueh-Ju, Chau -Yin Chen

**Affiliations:** 1 Department of Ophthalmology, Chang Gung Memorial Hospital, No. 6, West section, Chia-Pu RoadPu-Zih City, Chia-Yi County Taiwan; 2 College of Medicine, Chang Gung University, Taoyuan, Taiwan; 3 Department of Nursing, Chang Gung University of Science and Technology, Chiayi, Taiwan; 4 School of Traditional Chinese Medicine, College of Medicine, Chang Gung University, Taoyuan, Taiwan; 5 Department of Ophthalmology, Chang Gung Memorial Hospital, Linkou, Taiwan

**Keywords:** Chorioretinal folds, Hyperopic shift, Pleomorphic adenoma of the lacrimal gland (PALG), Optical coherence tomography angiography (OCT-A), Case report

## Abstract

**Background:**

Chorioretinal fold (CFs) is a rare condition resulting from undulations in the choriocapillaris, Bruch's membrane, retinal pigment epithelium and occasionally neurosensory retina. It can be idiopathic or due to different etiologies. The use of spectral-domain optical coherence tomography (SD-OCT) has increased the diagnosis of CFs and helped in differentiation from other etiologies. Recently, optical coherence tomography angiography (OCT-A) emerged as a non-invasive imaging technique allowing visualization of the individual layers of microvasculature of the retina and the choroid by comparing consecutive B-scans. We described a rare case of pleomorphic adenoma of the lacrimal gland (PALG) causing hyperopic shift and CFs with the new OCT-A technology, getting deeper insight into vascular changes of this disease.

**Case presentation:**

A 40-year-old Asian man experienced progressive blurred vision in his right eye over 6 months. The patient’s initial axial lengths were 25.55 mm in the right eye and 28.13 mm in the left eye. Fundus examination in the right eye revealed oblique CFs as well as the SD-OCT displayed. Magnetic resonance imaging showed intraconal mass extended from superior temporal side of the right orbit. The patient then received tumor removal surgery through lateral orbitotomy and histopathology confirmed a pleomorphic adenoma of the orbit.

The patient had regular follow-up for 1 year. His best corrected visual acuity markedly improved from 20/50 to 20/20 with nearly stationary AXL. We performed OCT-A at one year after the surgery, which showed early visualization of deep choroidal vessels. The scleral remodeling due to mass effect of retrobulbar tumor also caused displacement of the deep large choroidal vessels over the superior macular area even after tumor removal.

**Conclusions:**

We reported a rare case of PALG with hyperopic shift and CFs as initial presentation. Surgical removal of the tumor partially resolved the CFs and contributes to impressive visual acuity recovery. The use of OCT-A provided a deeper insight to vascular architecture changes resulting from scleral remodeling after long-term tumor compression.

## Background

Chorioretinal folds (CFs) is a rare condition resulting from undulations in the choriocapillaris, Bruch's membrane, retinal pigment epithelium (RPE) and occasionally neurosensory retina. It can be idiopathic or due to different etiologies such as orbital tumor, thyroid eye disease, orbital cellulitis, hypotony, scleritis, retinal detachment, trauma or even medication [1–5]. Symptoms from choroidal folds can vary from asymptomatic to hyperopic shift or metamorphopsia depending on their cause and the rapidity of their progression [6, 7]. The use of multimodal imaging such as spectral-domain optical coherence tomography (SD-OCT) has increased the diagnosis of CFs and helped in differentiation from other etiologies. Recently, optical coherence tomography angiography (OCT-A) emerged as a non-invasive imaging technique allowing visualization of the individual layers of microvasculature of the retina and the choroid by comparing consecutive B-scans [8]. We described a rare case of pleomorphic adenoma of the lacrimal gland (PALG) causing hyperopic shift and CFs with the new OCT-A technology, attempting to deeper insight into vascular changes at the level of Bruch's membrane and RPE complex, choriocapillaris and choroidal vessel layer after tumor removal.

## Case presentation

**Fig. 1:**
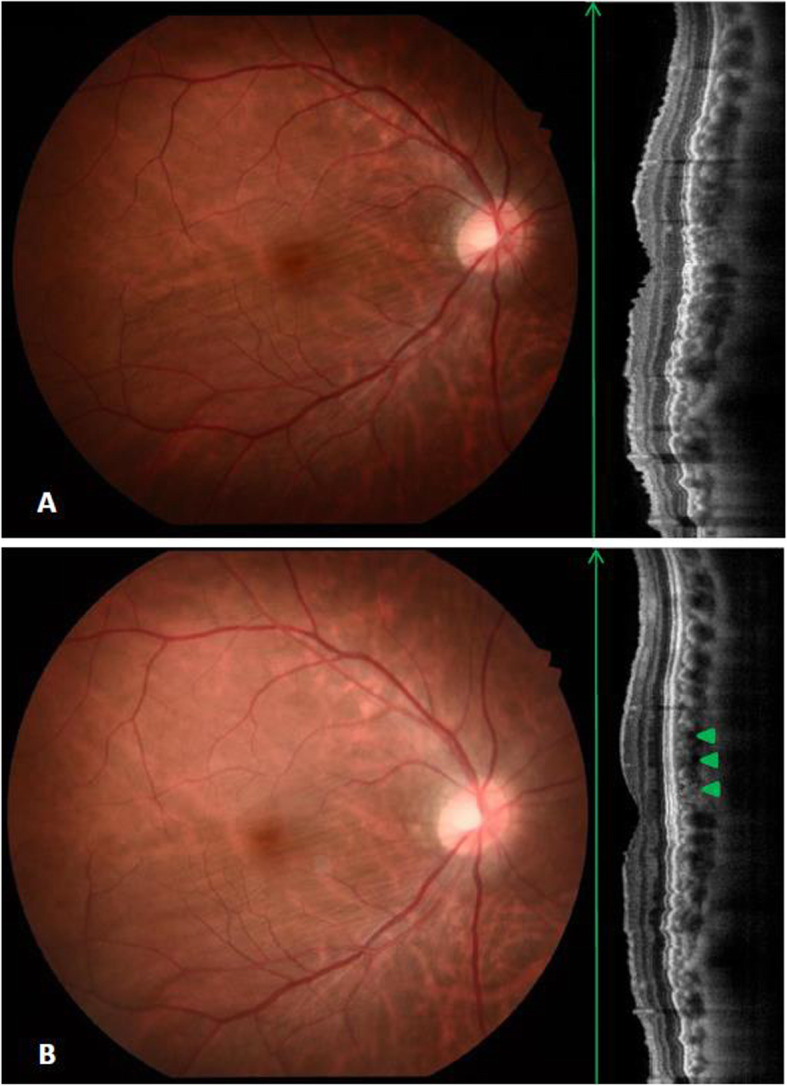
Color fundus and SD-OCT of the lesion eye before and after operation. (**a**) Oblique chorioretinal folds on fundus examination were in consistent with the SD-OCT displayed. (**b**) About 7 months after surgical removal of the tumor, follow up fundus photograph showed unchanged chorioretinal folds, while OCT revealed partial resolution of superior portion of macula (arrowhead)

A 40-year-old Asian man experienced progressive blurred vision in his right eye over 6 months. He had myopia with about -9.0 diopters on both eyes before. Upon examination, best corrected visual acuity (BCVA) was 20/50 in the right eye and 20/20 in the left eye with refractive error of -5.00 D and -9.25 D respectively. The patient’s initial axial lengths (AXL) were 25.55 mm in the right eye and 28.13 mm in the left eye. Fundus examination in the left eye proved to be unremarkable, while the right eye revealed oblique CFs as well as the SD-OCT displayed (Fig. 1a).

**Fig. 2:**
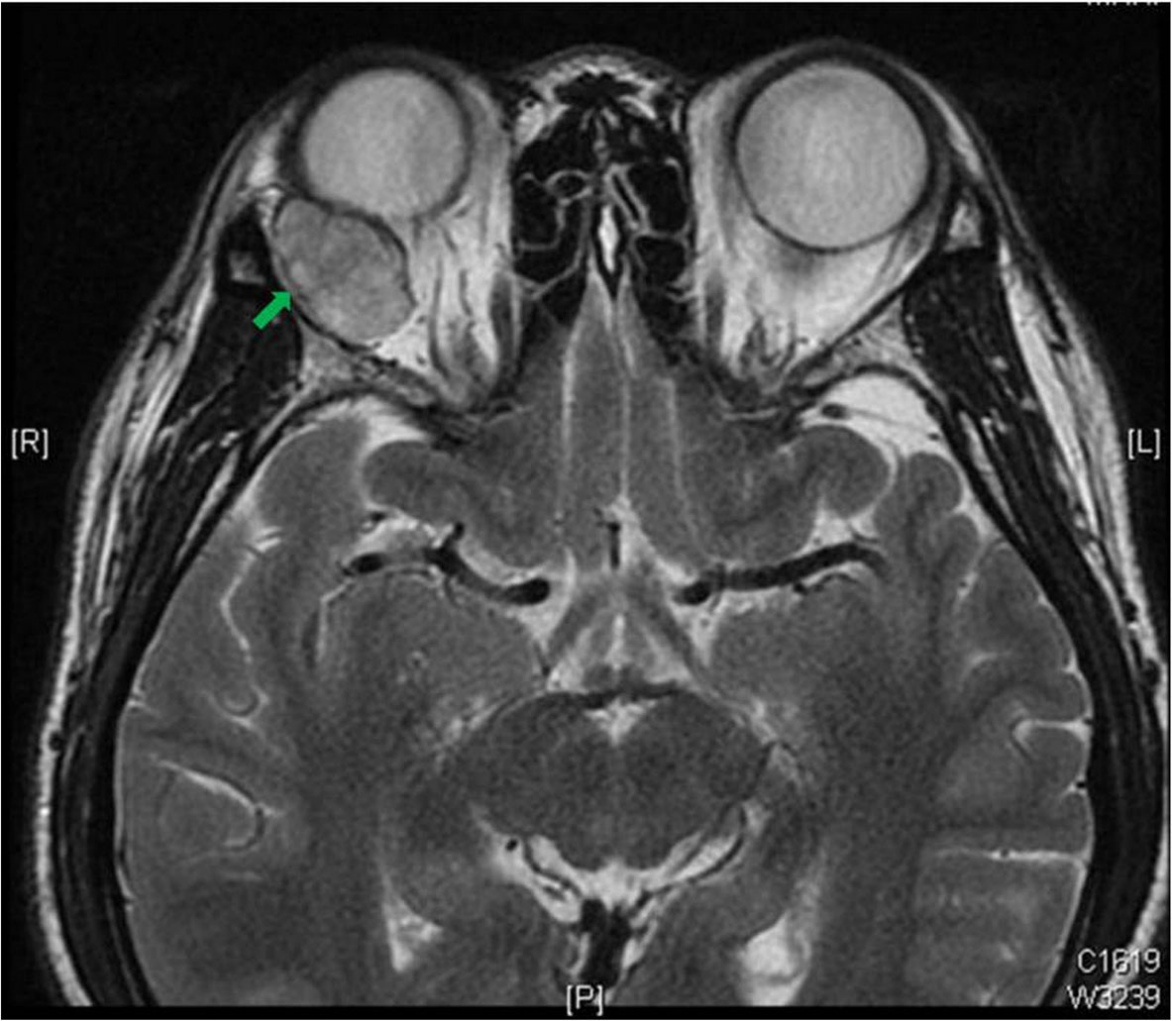
Magnetic resonance imaging (MRI) image of the patient. Magnetic resonance imaging (MRI), T2WI, axial view showed a well-defined, oval shape right intraconal tumor that hyperintensity signal with contrast enhancement about 16 × 24 × 22 mm in size (arrow)

Magnetic resonance imaging (MRI) showed a well circumscribed, homogenous, 16 × 24 × 22 mm large, oval-shaped intraconal mass extended from superior temporal side of the right orbit (Fig. 2). The patient then received removal of tumor through lateral orbitotomy and histopathology confirmed a pleomorphic adenoma of the orbit.

**Fig. 3:**
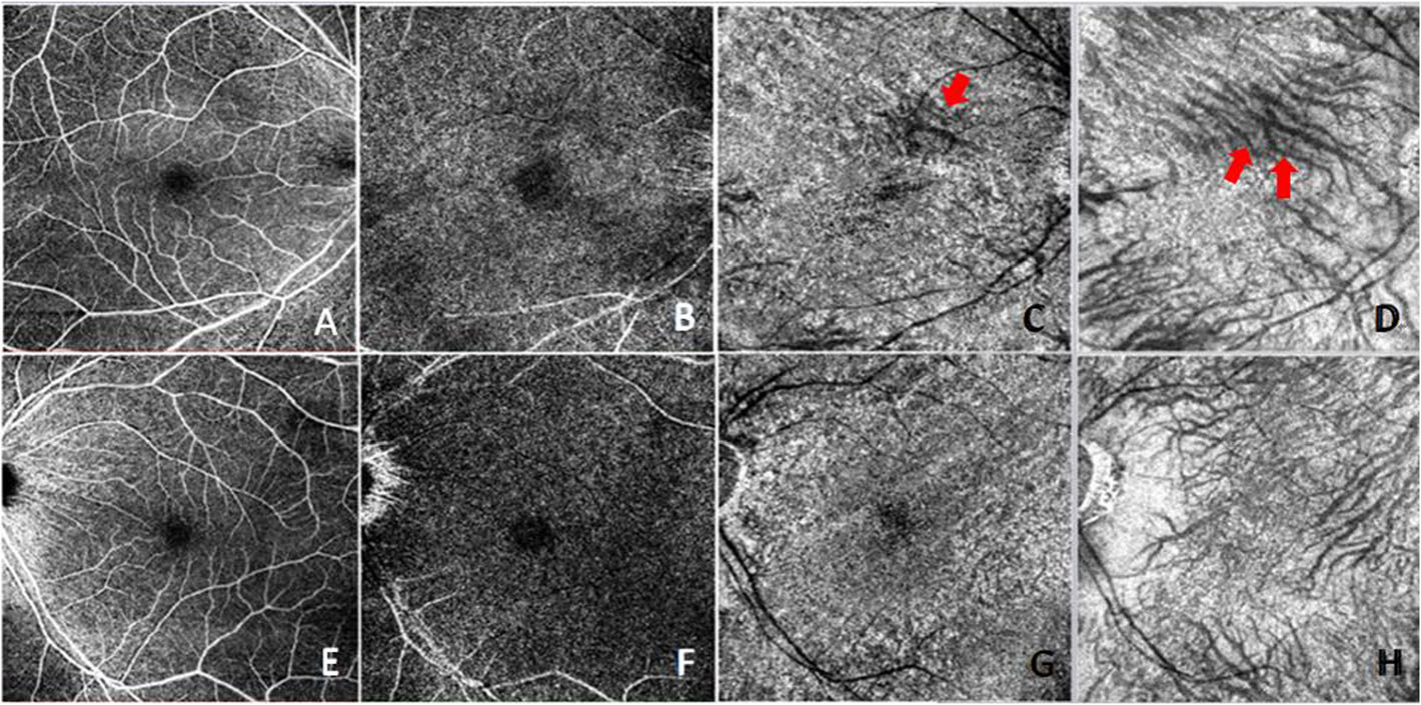
Optical coherence tomography angiography (OCT-A) of the patient one year after operation. Images **a**-**d** showed consecutive OCT-A scans of right eye and image **e**–**h** were left eye, which were superficial plexus, deep plexus, choriocapillaris and choroid layer respectively. Early visualization of deep choroidal vessels was demostrated (Fig. 3 C, arrow) and the scleral remodeling due to mass effect of retrobulbar tumor also caused displacement of the deep large choroidal vessels over the superior macular area even after tumor removal (Fig. 3 D, arrow)

The patient had regular follow-up for 1 year and the refraction, BCVA and AXL are showed in Table 1, which BCVA markedly improved to 20/20 with nearly stationary AXL. Follow up fundus examination showed unchanged CFs with OCT revealed partial resolution of superior portion of macula (Fig. 1b, arrowhead). We performed OCT-A at one year after the surgery. The OCT-A showed no alterations at the superficial plexus segmentation (Fig. 3a). In deep plexus, slabs of reduction of the signal were found around the fovea (Fig. 3b). In choriocapillaris and choroid layer, early visualization of deep choroidal vessels was demonstrated (Fig. 3c, arrow) with signal void areas in correspondence with the tumor location (superior side) rather than the area of chorioretinal folds (Fig. 3c, d). Furthermore, the scleral remodeling due to mass effect of retrobulbar tumor also caused displacement of the deep large choroidal vessels over the superior macular area even after tumor removal (Fig. 3d, arrow).

**Table 1:** Axial length measurement using IOL Master

Date	OD			OS		
	**Ref. RE**	**AXL**	**BCVA RE**	**Ref. LE**	**AXL**	**BCVA LE**
09/2018	-5.00/-0.75X20	25.55	20/50	-9.25/-1.25X175	28.13	20/20
01/2019	-4.50/-1.00X10	25.59	20/30	-9.00/-1.00X175	28.16	20/20
02/2019	-4.00/-1.00X8	25.68	20/20	-8.75/-0.75X174	28.14	20/20
03/2019	-4.75/-1.00X10	25.72	20/20	-9.00/-0.75X170	28.19	20/20
05/2019	-4.50/-0.75X12	25.75	20/20	-9.50/-0.75X173	28.18	20/20
07/2019	-4.25/-1.00X12	25.76	20/20	-8.50/-1.25X174	28.19	20/20

*Ref* Refraction, *BCVA* Best-corrected visual acuity, *RE* Right eye, *LE* Left eye, *AXL* Axial length

## Discussion

Refractive errors and CFs may arise as a result of the stresses placed on the globe and optic nerve by intra-orbital masses and usually resolves after surgical removal of the tumor [9]. However, several case reports documented the refractive errors or folds persist for even years after tumor removal [6, 10, 11]. The persistent flattening of the posterior pole and CFs has been attributed to scleral remodeling after long-standing compression. The pleomorphic adenoma of the lacrimal gland has a relative slow growth pattern, which the duration of the first symptom before diagnosis may be over a year [12]. Even the tumor was surgically removed the axial length of the eye did not revert to its original state in our case after long-standing compression, as well as the hyperopic shift of the lesion eye.

On the aspect of detailed anatomical level of choroid and retina vessels changes and the status of perfusion causing from mechanical stress, only few study has investigated. Del Turco et al. reported 3 cases of ocular CFs from different etiologies other than tumor-related using OCT-A and hypothesized blood flow alteration at the choriocapillaris level in correspondence of the fold [13]. In our case, instead of the features of transversal lines of rarefaction of retinal vessels result from chorioretinal folds, the choriocapillaris and choroidal layers revealed a patchy pattern of signal reduction with changes of deep choroidal vessel direction. We hypothesize that this change in the appearance of choroidal vessels direction could be due to intrusion of the choroid layer with scleral remodeling after long-term compression even with tumor removal.

The postoperative visual outcome and refraction alterations mainly depend on initial VA, the size of posterior tumor, degree of hyperopia induced and surgical manipulation of the optic nerve [9]. In the present case, the postoperative BCVA had excellent improvement to 20/20, which may attribute to partial resolving CFs after complete tumor removal. Compared to fundus examination only, OCT could better recognize subtle change of CFs and may aid in the diagnosis and the follow-up of the disease and OCT-A could further provide a more detail information about blood flow affected by different etiology causing CFs.

In summary, we reported a rare case of PALG with hyperopic shift and CFs as initial presentation. Surgical removal of the tumor partially resolved the CFs and contributes to impressive visual acuity recovery. The use of OCT-A provided a deeper insight to vascular architecture changes resulting from scleral remodeling after long-term tumor compression.

## Data Availability

All data generated or analyzed during this study are included in this published article.

## Abbreviations

CFs: Chorioretinal folds
RPE: Retinal pigment epithelium
SD-OCT: Spectral-domain optical coherence tomography
OCT-A: Optical coherence tomography angiography
PALG: Pleomorphic adenoma of the lacrimal gland
BCVA: Best corrected visual acuity
AXL: Axial lengths
MRI: Magnetic resonance imaging
